# Patient satisfaction and treatment adherence of stable human immunodeficiency virus-positive patients in antiretroviral adherence clubs and clinics

**DOI:** 10.4102/phcfm.v10i1.1759

**Published:** 2018-06-18

**Authors:** Gabi A. de Jager, Talitha Crowley, Tonya M. Esterhuizen

**Affiliations:** 1 Department of Nursing and Midwifery, Faculty of Medicine and Health Sciences, Stellenbosch University, South Africa; 2 Division of Epidemiology and Biostatistics, Department of Global Health, Faculty of Medicine and Health Sciences, Stellenbosch University, South Africa

## Abstract

**Background:**

South Africa has experienced a substantial increase in access to antiretroviral treatment (ART) in recent years. Effective strategies to manage access to treatment need to be incorporated into and implemented in ART programmes. Antiretroviral treatment adherence clubs are a new strategy that is being implemented in various parts of South Africa.

**Aim:**

The aim of the study was to investigate treatment adherence and patient satisfaction of stable human immunodeficiency virus (HIV) patients on ART in ART adherence clubs and clinics.

**Setting:**

The study was conducted in the Eden district of the Western Cape, South Africa.

**Methods:**

A cross-sectional analytical study was conducted to examine the relationships between patient satisfaction and treatment adherence in ART adherence clubs and clinics in the Eden district, Western Cape province, South Africa. Validated questionnaires were used to measure patient satisfaction and self-reported treatment adherence.

**Results:**

The study included 320 participants (98 club and 222 clinic) from 13 primary health care clinics. The analyses showed that higher levels of satisfaction could be predicted with club participants compared to clinic participants (*p* = 0.05). There was no significant difference between clinic and club participants with regards to treatment adherence. However, being adherent was more likely in participants who were satisfied (odds ratio = 3.18, 95% confidence interval [1.14–7.11], *p* < 0.01).

**Conclusion:**

Antiretroviral treatment adherence clubs provide a service that patients are more satisfied with although they are not more adherent to treatment. This strategy may be effective for the delivery of long-term care for patients on ART.

## Introduction and background

An estimated 36.7 million people in the world live with the human immunodeficiency virus (HIV), with 20 million accessing treatment globally.^1^ From 2011 to 2012, 2.3 million people in sub-Saharan Africa were added to HIV and/or acquired immune deficiency syndrome (AIDS) programmes and South Africa experienced a 75% increase in access to antiretroviral treatment (ART).^2^ Effective strategies need to be incorporated into and implemented in ART programmes to help alleviate the pressure on health care services.^3^

South Africa has the largest population of people living with HIV in the world, with estimated numbers standing at 7 million people.^4^ Antiretroviral treatment guidelines state that all HIV-positive patients, irrespective of their cluster of differentiation 4 (CD4) cell count, are eligible for ART.^5^ Changes in treatment guidelines have led to a marked increase in new patients receiving ART, with numbers in excess of 300 000 annually.^6^

Health care systems with health care worker shortages, especially medical practitioners and nurses, face significant challenges in continuing the provision of ART to rapidly growing numbers of patients.^3,7^ A systematic review conducted in 2007 indicated that in sub-Saharan Africa only 60% of patients remained on ART 2 years after starting treatment, with loss to follow-up and death as the leading causes of patient attrition.^8^ It is with these two challenges in mind that various new strategies are being investigated to alleviate the strain on the national health care system and improve retention in care of ART patients.

These strategies include task shifting of HIV treatment and the decentralisation of ART to primary health care (PHC) clinics and community ART adherence groups. Task shifting allows for specific care tasks to be moved from highly qualified health care workers, for example, doctors to health care workers with shorter training and fewer qualifications, such as nurses.^9^ Decentralisation entails moving ART delivery from hospitals to more peripheral health care facilities, such as clinics, and even into communities.^10^ These two strategies have provided essential relief and assistance for health care providers and assisted in increasing access to ART for patients. However, high rates of patient attrition remain, and it is argued that service delivery should be expanded to the community.^11,12^ Ford and Mills recommend that care be made available as close as possible to a patient’s home and that it be as minimally intrusive as possible to the patient’s life, as is the case with ART adherence clubs.^12^

The World Health Organization (WHO) promotes patient support groups as an intervention to address poor adherence and improve retention in care.^13^ Patient support groups are an essential mechanism of service delivery in providing patients with health education and peer support within the community.^14^ Luque-Fernandez et al. found that patient support groups, such as ART adherence clubs, were an effective model for improving retention in care and documented viral suppression rates.^14^

Antiretroviral treatment adherence clubs consist of 15–30 stable ART patients who meet as a group every 2 months, either at the PHC clinic outside of busy times or in the community. A stable ART patient is defined as someone who has been on ART for at least 12 months and who has had two recent undetectable consecutive viral loads (< 400 copies/mL).^15^ The ART adherence club is facilitated by a non-clinical staff member (counsellor), and medicines are prepacked for each person and brought to the club by the counsellor.

A study on ART adherence clubs in a South African urban setting showed that ART adherence clubs improved retention in care and that more patients in the ART adherence club were viral load suppressed compared to primary health care (PHC) clinic patients.^14^ The Western Cape Department of Health, South Africa, has adopted the guidelines for ART adherence clubs suggested by Luque-Fernandez and colleagues as a means to improve retention in care and adherence.^16^

Treatment adherence is the extent to which a patient’s behaviour (taking medication, following a diet and/or effecting lifestyle changes) corresponds with agreed recommendations from a health care provider.^17^ With ART, adherence to treatment is critical to ensure that the life-extending benefits of ART are maximised and viral suppression is achieved.^18^ According to Dang et al., treatment adherence is positively influenced by overall patient satisfaction.^19^ Patient satisfaction is defined as a patient’s general orientation towards a total experience of health care.^20^ Satisfied patients are likely to be an indication of a successful HIV programme with larger numbers of adherent patients.^19,20^

### Problem statement

Antiretroviral treatment adherence clubs are an effective model of care for stable adult ART patients in an urban setting.^14^ In the Western Cape, South Africa, this strategy has been fully implemented. Patient satisfaction and the effectiveness of ART adherence clubs have not been scientifically evaluated in other settings. The Eden district, a rural district of the Western Cape, has 14 757 patients on ART.^21^ Of these, 1747 patients are receiving care in ART adherence clubs. No study had previously been undertaken in the Eden district to evaluate patient satisfaction and ART adherence in PHC clinics or ART adherence clubs. The literature asserts that as ART adherence clubs may be more convenient for patients (outside busy clinic times and in the community), they may improve patient satisfaction, leading to improved adherence. There is; however, no scientific evidence in the South African context to support this assumption.

### Aim

The aim of the study was to investigate treatment adherence and patient satisfaction of stable patients living with HIV on ART in ART adherence clubs and clinics.

## Methods

### Research design

As ART adherence clubs are a new model of care in the implementation phase, a quantitative analytical cross-sectional study was used.

### Population and sample

The population (*N* = 14 757) included stable patients living with HIV on ART receiving care either in ART adherence clubs or in PHC clinics in the Eden district of the Western Cape.

A multistage sampling strategy was used. According to the Eden district Health Department, there are 38 PHC clinics that provide ART care in the district. After communicating with the clinic managers, the researcher confirmed that there were only seven clinics that provided ART care in both ART adherence clubs and in the PHC setting. The researcher purposefully selected these seven clinics that had established ART adherence clubs at least six months prior to the start of the study. In these clinics, the researcher recruited a comparable number of participants attending the adherence club (*n* = 98) and participants attending the clinic (*n* = 106). One of these seven clinics was used for the pilot study in which 34 patients were recruited, 17 attending the club and 17 attending the clinic. An additional seven clinics without ART adherence clubs were randomly selected with the use of a computer program from the remaining clinics in the district. In these clinics, only participants attending the clinic were selected (*n* = 116). The reason participants from clinics with no ART adherence clubs were included was to account for the possible effect that ART adherence clubs might have on the clinic, for example, reducing the number of patients attending the clinic. The study, therefore, included 13 PHC clinics as one of the clinics was used for the pilot study and the data were not included in the main study.

Consecutive sampling was used to select participants as accurate up-to-date patient lists could not be obtained from either the ART adherence clubs or the clinics, making random sampling impossible. Patients attending the clinics or clubs were, therefore, asked to participate in the study when they attended the clinics. We assumed the order of patient presentation was random and thus random subsamples from each site were generated in this manner. In order to control for bias, the researcher recruited participants on different days and at different times throughout the clinics’ operating hours to ensure that the population was well represented. The sample included clinic patients (*n* = 222) and ART adherence club patients (*n* = 98).

The initial estimated required sample size was 105 clinic patients and 105 club patients (with a 95% confidence level and 80% power). This was estimated using the Luque-Fernandez et al.^14^ study, which found a 12% difference in retention in care (used as a proxy for adherence) between clinics and ART adherence clubs. The statistician confirmed that the actual study sample size had sufficient power to detect a difference between groups.

For inclusion in the study, patients had to be 18 years and older, HIV infected, on ART for at least 12 months and attending the clinic or club for at least six months. All pregnant patients were excluded as they would need to attend the clinic more frequently during pregnancy.

### Instrumentation

Data were collected using a researcher-administered questionnaire. Questionnaires with established validity and reliability were used to formulate the questionnaire: the Chaiyachati Patient Adherence Questionnaire by Chaiyachati et al.^18^ and the Patient Satisfaction with ART Services Questionnaire by Wouters et al.^20^ Both questionnaires had been used before in a rural South African PHC setting similar to that of the Eden district of the Western Cape. Basic biographical information was obtained in Section A. The first question determined the type of visit (clinic or club), followed by nine biographical questions and three questions regarding HIV and ART.

Section B consisted of five questions regarding treatment adherence. The five questions were made up of four closed-ended questions and one visual analogue scale question. A Likert scale was used to report the last missed dose and estimated adherence over the preceding 30 days. The questions related to the last missed dose and estimated adherence were dichotomised to represent adherence or non-adherence in order to conduct further analysis. Any response less than ‘never’ or ‘excellent’ was considered non-adherent. This option was chosen as it had the best combination of sensitivity and specificity to detect treatment failure. Chaiyachati et al. found that the estimated adherence over the preceding 30 days item performed the best to detect virologic and immunologic failure, with sensitivity and/or specificity of 92/3% and 100/5%, respectively (when defining non-adherence as self-reported adherence less than ‘excellent’).^18^ Non-adherence was coded as 0 and adherence as 1.

Section C consisted of 11 questions regarding patient satisfaction. A five-point Likert scale was used for 10 of these questions and followed by one open-ended question for patient complaints or compliments. This section of the questionnaire used a five-point Likert scale with options consisting of very satisfied = 5, satisfied = 2, neither satisfied nor dissatisfied = 3, dissatisfied = 2 and very dissatisfied = 1. A higher score indicated satisfaction.

The questionnaire was available in English, Afrikaans and Xhosa after being translated by experienced translators. The researcher collected the participants’ viral load results from their folders.

### Pilot study

A pilot study was conducted at one of the seven clinics that had ART adherence clubs with 34 participants (17 clinic and 17 club). From this group of participants, interim statistical analysis was performed, and minor changes were made to the questionnaire. These data were excluded from the study analysis.

### Data collection

The researcher trained three fieldworkers to assist with the data collection. The fieldworkers were fluent in all three languages. Patients were invited to participate after having received their treatment. The fieldworkers administered the questionnaires individually in a private consultation room at each of the respective clinics, and these individual sessions lasted approximately 10 min.

### Data analysis

The data collected were first prepared for analysis by being entered into Microsoft Excel. The data were then cross-checked by the researcher for any errors and omissions. Once the data had been checked, they were analysed with statistical software programs, IBM SPSS version 22^22^ and Stata version 14.^23^

Descriptive statistics (measures of central tendency and variance) were used to initially describe the data. Where there was missing data, the denominator used was the total non-missing per group. Patient satisfaction was measured by summing up responses to the 10 items measuring patient satisfaction and averaging the total, with a higher score meaning greater satisfaction. Missing values for the items making up the total satisfaction score were not imputed as it was assumed that they were random (10.1% missing total satisfaction scores for club participants and 13.3% for clinic participants). The following statistical analyses were performed: chi-squared analysis, independent *t*-test, bivariate correlations, multiple linear and logistic regression analysis. Where the assumptions of parametric tests were not met, non-parametric tests were conducted.

Multiple linear regression analysis was used to assess whether club or clinic attendance could predict patient satisfaction whilst adjusting for confounding variables. Logistic regression was used to assess whether the type of attendance could predict treatment adherence and viral suppression. These analyses controlled for clustering by facility using robust standard errors. For all models, bivariate associations between potential confounders and the outcomes were first assessed at the significance level of 0.1. All confounders that were associated with the outcome and the exposure of interest were entered into the model, and a backwards stepwise method was used to arrive at the final model, with removal probability set at 0.05. Likelihood ratio tests were conducted between each model to ensure validity at each stage of model fitting.

### Validity and reliability

The content validity of the data collection instrument was ensured by using established instruments. Both these questionnaires are regularly used in South Africa with HIV-positive patients on ART. In addition, two local experts in the field of HIV management were consulted to ensure the content validity of the instruments. The face validity and readability of the questionnaire were assessed during the pilot study. The reliability of the data collection instrument was ensured by using established questionnaires from the literature in which stability and equivalent reliability had been established. The Cronbach’s alpha coefficient was used to calculate the internal consistency of the patient satisfaction scale in the instrument. The Cronbach’s alpha was 0.78, an acceptable level^24^ and similar to reliability measures reported by Wouters et al.^20^

## Ethical considerations

The Health Research Ethics Committee at Stellenbosch University approved the study (S14/03/055). Further approval was obtained from the Western Cape Department of Health Ethics Committee (RP 101/2014). The researcher obtained permission from the Eden district municipal management, and each clinic manager personally granted permission for the research to be conducted in the respective clinics. Participants were provided with information about the study, participation was by choice and withdrawal from the study at any time was permitted without any discrimination. Each participant signed a consent form that was kept separate from the questionnaire. Questionnaires did not contain any patient indicators. All research documents are being kept in a locked filing cabinet for five years and will then be destroyed

## Results

### Biographical data

Three hundred and twenty participants were recruited for the study, of which 98 (30.6%) were from adherence clubs. The biographical data of the participants are depicted in Table 1. The majority of the participants were female (*n* = 241, 76.3%, Table 1). There was a significant difference between the clinic and club group in terms of language (X^2^ [2] = 6.96, *p* = 0.03). Xhosa-speaking participants were more likely to be in a club, and Afrikaans-speaking participants were more likely to be in a clinic. More than half of the participants were unemployed (*n* = 167, 52.2%), with participants in the clubs more likely to be employed full time or part time. The participants in the clinic were more likely to be unemployed. Table 1 shows that the majority of the participants had no other illnesses (*n* = 232, 72.5%). The majority of the participants reported feeling well on the day (*n* = 280, 87.8%).

**TABLE 1:** Biographical data.

Variable	Clinic					Club					Total					*p*
	*n*	Valid %	Mean	Median	SD	*n*	Valid %	Mean	Median	SD	*n*	Valid %	Mean	Median	SD	
**Gender**																0.83
Men	51	23.4	-	-	-	24	24.5	-	-	-	75	23.7	-	-	-	-
Women	167	76.6	-	-	-	74	75.5	-	-	-	241	76.3	-	-	-	-
Total	218	100	-	-	-	98	100.0	-	-	-	316	100.0	-	-	-	-
**Language**																0.03
Xhosa	98	44.2	-	-	-	57	58.1	-	-	-	155	48.4	-	-	-	-
English	40	18.0	-	-	-	18	18.4	-	-	-	58	18.2	-	-	-	-
Afrikaans	84	37.8	-	-	-	23	23.5	-	-	-	107	33.4	-	-	-	-
Total	222	100.0	-	-	-	98	100.0	-	-	-	320	100.0	-	-	-	-
**Employment**																< 0.01
Full- time	63	28.4	-	-	-	40	40.8	-	-	-	103	32.2	-	-	-	-
Part- time	30	13.5	-	-	-	20	20.4	-	-	-	50	15.6	-	-	-	-
Unemployed	129	58.1	-	-	-	38	38.8	-	-	-	167	52.2	-	-	-	-
Total	222	100.0	-	-	-	98	100.0	-	-	-	320	100.0	-	-	-	-
**Number of other illnesses**																0.08
No other illnesses	155	69.8	-	-	-	77	78.6	-	-	-	232	72.5	-	-	-	-
One other illness	56	25.2	-	-	-	20	20.4	-	-	-	76	23.8	-	-	-	-
Two other illnesses	10	4.5	-	-	-	1	1.0	-	-	-	11	3.4	-	-	-	-
Three or more other illnesses	1	0.5	-	-	-	0	0.0	-	-	-	1	0.3	-	-	-	-
Total	222	100.0	-	-	-	98	100.0	-	-	-	320	100.0	-	-	-	-
**Feeling well**																0.27
No	30	13.6	-	-	-	9	9.2	-	-	-	39	12.2	-	-	-	-
Yes	191	86.4	-	-	-	89	90.8	-	-	-	280	87.8	-	-	-	-
Total	221	100.0	-	-	-	98	100.0	-	-	-	319	100.0	-	-	-	-
Age	221	100.0	39.4	40.0	9.2	95	100.0	39.4	39.0	9.4	316	100.0	39.4	39.0	9.3	0.99
Educational level	217	100.0	9.2	10.0	2.8	98	100.0	9.1	10.0	2.7	315	100.0	9.2	10.0	2.8	0.90
Monthly income	214	100.0	1034.5	0.0	1331.8	98	100.0	1617	1200.0	2088.8	312	100.0	1217.4	500.0	1627.6	0.02
Number of pills taken	222	100.0	3.8	3.0	3.2	98	100.0	3.4	3.0	2.3	320	100.0	3.7	3.0	2.9	0.44

The mean age of participants was 39.4 (standard deviation [SD] 9.3 years). The median grade completed at school was Grade 10. There was a significant difference between the clinic and club group in terms of monthly income (Mann–Whitney U, *p* = 0.02). The median monthly income in the club group was higher than in the clinic group. The mean number of pills taken was 3.7, and the median was 3. The highest number of pills taken was 30.

### Human immunodeficiency virus-related data

HIV-related data obtained from participants are shown in Table 2. The two most recent viral loads were combined to create an average viral load, and this variable was dichotomised to report viral suppression. A participant was classified as viral load suppressed if the average viral load was less than 400 copies/mL. The majority of the participants had a suppressed viral load (*n* = 259, 84.4%). There was a significant difference between the clinic and club group with regard to viral load suppression (X^2^ [1] = 22.55, *p* < 0.001). The portion of participants who were viral load suppressed in the club group was 98.9% compared to 77.7% in the clinic group. The mean log viral load was 2.35 (SD 0.80). The majority of participants (48.9%, *n* = 140) had been diagnosed with HIV 1–5 years previously. The mean number of years on ART was 4.8.

**TABLE 2:** Human immunodeficiency virus-related data.

Variable	Clinic					Club					Total					*p*
	*n*	Valid %	Mean	Median	SD	*n*	Valid %	Mean	Median	SD	*n*	Valid %	Mean	Median	SD	
**Viral suppression**																< 0.001
Yes	164	77.7	-	-	-	95	98.9	-	-	-	259	84.4	-	-	-	-
No	47	22.3	-	-	-	1	1.1	-	-	-	48	15.6	-	-	-	-
Total	211	100.0	-	-	-	96	100.0	-	-	-	307	100.0	-	-	-	-
**Average log viral load**	212	100.0	2.48		0.93	96	100.0	2.08	-	0.25	308	100.0	2.35	-	0.80	0.280
**Human immunodeficiency virus diagnosis**																0.100
1–5 years ago	96	33.6	-	-	-	44	47.3	-	-	-	140	48.9	-	-	-	-
6–10 years ago	73	25.5	-	-	-	44	47.3	-	-	-	117	40.9	-	-	-	-
More than 10 years ago	24	8.4	-	-	-	5	5.4	-	-	-	29	10.1	-	-	-	-
Total	193	100.0	-	-	-	93	100.0	-	-	-	286	100.0	-	-	-	-
**Years on treatment**	221	100.0	4.5	4.0	2.3	98	100.0	5.4	5	2.4	319	100.0	4.8	4.3	2.4	< 0.010

### Treatment adherence

There was a significant difference between the clinic and club group with regard to the last missed dose (Fisher’s exact = 18.671, *p* < 0.01) but not when comparing estimated adherence over the preceding 30 days (Table 3). The other three measures for adherence, namely; ‘total missed doses over the preceding seven days’, ‘total missed doses taken late’ and the visual analogue scale ‘percentage of pills taken in the last month’, did not show significant differences between the two groups.

**TABLE 3:** Treatment adherence: Likert-scale items.

Variable	Clinic		Club		Total		*p*
	*n*	Valid %	*n*	Valid %	*n*	Valid %	
**Last missed dose**							< 0.01
Within the week	22	9.9	9	9.2	31	9.7	-
1–2 weeks ago	16	7.2	4	4.1	20	6.3	-
2–4 weeks ago	6	2.7	4	4.1	10	3.1	-
1–3 months ago	14	6.3	5	5.1	19	5.9	-
More than 3 months ago	25	11.3	0	0.0	25	7.8	-
Never	139	62.6	76	77.6	215	67.2	-
Total	222	100.0	98	100.0	320	100.0	-
**Estimated adherence in last 30 days**							0.66
Very poor	3	1.4	0	0.0	3	1.4	-
Poor	3	1.4	0	0.0	3	1.4	-
Fair	7	3.2	1	1.0	8	2.5	-
Good	33	14.9	14	14.3	47	14.7	-
Very good	57	25.7	30	30.6	87	27.2	-
Excellent	119	53.6	53	54.1	172	53.8	-
Total	222	100.0	98	100.0	320	100.0	-

The number of pills taken per day was significantly lower in patients who were categorised as adherent as measured by the last missed dose adherence question (Mann–Whitney U, *p* = 0.001) and the estimated adherence question (Mann–Whitney U, *p* = 0.02).

### Satisfaction

The mean satisfaction score was 4.5 (SD 0.5, range 1.7–5.0) and the median 4.6, indicating that most patients were very satisfied or satisfied with the service they were receiving. The item with the lowest mean score related to waiting time (Mean = 3.5, SD 1.5), indicating that participants were the least satisfied with waiting time. Participants attending clubs were significantly more satisfied with waiting time (X^2^ [4] = 74.94, *p* < 0.001).

### Regression analyses

#### Treatment adherence

Estimated adherence over the preceding 30 days was used first to create a model (Table 4). There was no significant difference between clinic and club participants with regard to estimated adherence (OR = 0.80, *p* = 0.57) after controlling for language and satisfaction. Being adherent was more likely in participants who were satisfied (OR = 2.08, *p* < 0.01, 95% CI 1.24–3.5). For every one unit increase in the satisfaction score, adherence increases 2.08 times. The explained variance in estimated adherence was low with this model as indicated by the *R*^2^ of 0.05.

**TABLE 4:** Logistic regression model for estimated adherence (adherent vs. non-adherent).

Estimated adherence	Odds ratio	*p*	95% Confidence interval
Attendance: Club	0.80	0.57	0.36 – 1.75
**Language**†			
Afrikaans	0.42	< 0.01	0.25 – 0.69
English	0.93	0.83	0.48 – 1.78
Satisfaction mean score	2.08	< 0.01	1.24 – 3.50

Note: Estimated adherence in last 30 days (*n* = 285, adjusted for 13 clusters in clinic; pseudo *R*^2^ = 0.05).

† , Xhosa used as the reference group.

Another measure of adherence, namely, the last missed dose, was used to create a second model (Table 5). Non-adherence was coded as 0 and adherence as 1. The last missed dose revealed no significant difference between clinic and club participants. Being adherent was more likely in participants who were satisfied (OR = 3.18, *p* < 0.01, 95% CI 1.14–7.11) after adjusting for language, years on ART and viral suppression. For every one unit increase in the satisfaction score, the odds of being adherent increased threefold. The explained variance (16.3%) in this model was higher than in the first model (*R*^2^ of 0.05 [5%]).

**TABLE 5:** Logistic regression model on adherence (last missed dose – adherent vs. non-adherent).

Adherence	Odds ratio	*p*	95% Confidence interval
Attendance: Club	1.30	0.49	0.60– 2.82
**Language**†			
Afrikaans	0.20	0.01	0.06 – 0.68
English	0.32	0.02	0.12 – 0.86
Years on ART	0.85	< 0.01	0.75 – 0.95
Satisfaction mean score	3.18	< 0.01	1.14 – 7.11
Viral suppression: Yes	0.48	0.02	0.26 – 0.87

Note: Adherence (last missed dose) (*n* = 274, adjusted for 13 clusters in clinic; pseudo *R*^2^ = 0.1625).

ART, antiretroviral treatment.

† , Xhosa used as the reference group.

#### Patient satisfaction

Table 6 indicates that higher levels of satisfaction were predicted with club attendance than with clinic attendance after adjusting for the presence or contribution of every other variable in the model (*β* = 0.22, *p* = 0.05, 95% CI −0.004 – 0.444). Although the association is marginal, it indicates that patients attending clubs are at least as satisfied with the service, if not more, than those attending regular clinic services. The total variance explained was 21.2% (*R*^2^ = 0.212).

**TABLE 6:** Multiple regression model for mean satisfaction score.

Satisfaction	Coefficient	*p*	95% Confidence interval
Attendance: Club	0.22	0.05	−0.004 – 0.444
**Language**†			
Afrikaans	0.11	0.13	−0.040 – 0.28
English	0.14	< 0.01	0.040 – 0.25
Number of other illnesses	−0.11	0.03	−0.190 – −0.01
Years on ART	0.04	0.02	0.010 – 0.07
Last missed dose	0.04	< 0.01	0.170 – 0.41
Feeling well: No	−0.32	0.03	−0.620 – −0.03
Viral suppression: No	0.16	0.07	−0.010 – 0.32

Note: Satisfaction mean score *n* = 274, adjusted for 13 clusters in clinic; *R*^2^ = 0.212.

ART, antiretroviral treatment.

† , Xhosa used as the reference group.

## Discussion

The findings for treatment adherence showed that the majority of the participants in both groups were adherent. However, the club group had slightly better adherence levels with both the last missed dose (club – 77.6% vs. clinic – 62.6%) and estimated adherence (club – 54.1% vs. clinic – 53.6%). Despite high levels of self-reported adherence, 15.6% of participants were found not to have suppressed viral loads. This is high in comparison to a study by Chaiyachati et al. in which 9% of participants had unsuppressed viral loads. This supports suggestions that self-reported adherence performs poorly in identifying treatment failure.^18^

The results showed that a large pill burden decreased treatment adherence, which confirms the findings of an earlier study by Chesney.^25^ The implementation of fixed-dose combination pills (single-pill combination) in South Africa reduces the pill burden for HIV-positive patients,^5^ but the pill burden of patients living with HIV may increase owing to the increasing comorbidity of other chronic illnesses.

The majority of the participants were satisfied with the care they received in both the ART adherence clubs and the PHC clinics. This corresponds with satisfaction studies conducted in South Africa, specifically with HIV and/or AIDS programmes that concluded that, in general, patients were satisfied with the care they were receiving.^20,26,27^ In general, the findings suggest that HIV-positive patients are satisfied with the service and/or care that they receive.

The study findings showed that the PHC clinic participants were less satisfied with waiting time. This is supported by previous studies.^20,27,28^ For policy-makers and managers, this information is valuable as studies conducted on patient support groups for HIV-positive patients reported that patient support groups reduced the waiting time for patients.^7^ With specific reference to the South African model, it is argued that the ART adherence clubs had better outcomes as a result of shorter waiting times.^14^ This supports the finding that the ART adherence club group was significantly more satisfied than the PHC clinic group with regard to waiting time and should not be overlooked.

Antiretroviral treatment adherence clubs meet outside of busy clinic times, which are generally early mornings or late afternoons, as this is convenient for working patients.^14^ The demographic data support this as the club group had a higher percentage of employed participants. The total number of full-time and part-time employed participants was 61% compared to 42% in the PHC clinic group. The difference between the ART adherence club and the PHC clinic group specifically referring to hours that the facility is open is supported by this finding. Clubs that meet outside of busy clinic times are very attractive and convenient for burdened health care workers as this decongests clinics. Employed patients are often conflicted by regular work attendance and inconvenient clinic times. For these patients, missing work regularly to attend the clinic for treatment may result in less pay and possibly retrenchment. Antiretroviral treatment adherence clubs provide patients with a workable solution to this real problem.

The club group in this study had a lower percentage of non-viral suppression (*n* = 1, 1%) compared to the percentage reported in Luque-Fernandez et al.^14^ (*n* = 14, 3%). However, in the clinic group, non-viral suppression was higher in this study (*n* = 47, 22.3%) than in that of Luque-Fernandez et al. (*n* = 214, 9%).^14^ This is consistent with another South African study that found that urban residents were almost three times more adherent than rural residents.^29^ There may be unique challenges to adherence in the rural setting that require further investigation.

The regression model for patient satisfaction showed significant differences for language, number of other illnesses, years on ART and feeling well or unwell. With regard to feeling well or unwell, some support from the literature was found. A 2002 study on patient experiences and satisfaction with health care found an association between satisfaction and health status, similar to feeling well or unwell.^30^ This study suggests that feeling well or unwell does influence a patient’s experience of care and services received, but the correlation is weak.^30^ No other literature to clarify and support the association between patient satisfaction and language, number of illnesses and years on ART could be found at the time of review.

The treatment adherence regression models found that patient satisfaction did influence treatment adherence. This confirms what previous studies have shown: satisfied patients are more likely to be adherent.^20,31,32^

The above provides further evidence that ART adherence clubs may affect HIV outcomes positively because satisfied ART patients are more likely to be adherent to treatment. A systematic review found five studies reporting that increased adherence was associated with patient support group participation such as ART adherence clubs.^13^ Antiretroviral treatment adherence clubs provide a practical measure for HIV and/or AIDS programmes to improve and maintain adherence of stable patients living with HIV that should be implemented into more HIV and/or AIDS programmes. HIV/AIDS syndrome programmes could improve treatment adherence by improving patients’ experience of care and services received with the use of patient satisfaction surveys.

### Strengths and limitations

The cross-sectional design of this study provides HIV and / or AIDS programme developers and managers with valuable information for the club model of care in a rural setting. The sample of the study was large enough to detect significant differences between the groups. The sample was collected from 13 clinics from different towns in the Eden district, thereby increasing the representativeness of the study and its generalisability to other areas and population groups in South Africa.

A potential shortfall of this study may be the causal nature of adherence. The club participants are more likely to be adherent as adherence is a requirement for club inclusion. This would create a false-positive adherence in the club participants for the study. As this form of bias remains largely uncontrollable, the researcher attempted to curb this by using participants with a minimum attendance of six months.

Self-report measures of treatment adherence by patients proved to be another important potential shortfall as patients may have overestimated their own adherence. The researcher attempted to allow for this by using established adherence measures.

The researcher discovered that in the translation to Afrikaans and Xhosa, certain concepts in the questionnaire may have lost their meaning or may have been understood differently in the different languages. The misunderstanding of certain concepts, specifically with regard to treatment adherence and patient satisfaction, may have influenced the study findings to a certain degree. This was seen in the regression analyses that showed significant differences between Xhosa and English with the mean satisfaction score. Both treatment adherence regression models revealed differences between Xhosa and English and Xhosa and Afrikaans with regard to adherence. These differences could be interpreted as concepts in the questionnaire being understood differently in the three languages.

## Conclusion

Antiretroviral treatment adherence clubs provide a service where patients are more satisfied although their reported adherence was not significantly different from that of patients attending clinics. This strategy may be effective for the delivery of long-term care for patients on ART. The comparison of treatment adherence and patient satisfaction of patients living with AIDS revealed more than just the differences between ART adherence clubs and PHC clinics. The evidence supports the relationship between patient satisfaction and treatment adherence, irrespective of whether they attend the club or the clinic. The study has added to the empirical evidence that ART adherence clubs are an effective model for delivering ART to the ever-growing population of people living with HIV.
